# On Black and Green Teas

**Published:** 1853-07

**Authors:** 


*On Black and Green Teas.*—Though the distinct preparation of green and black tea from the respective plants, the *Thea viridis* and the *Thea Bohea,* has been warmly advocated by many botanists, yet it is now generally admitted by all parties, that green and black teas can be and are made, indiscriminately from the same parcel of leaves, taken from the same species of plant. It is nevertheless, well known to all persons, that the infusions from these teas have marked differences in color and flavour, and that the effects produced in certain constitutions by green tea, such as nervous irritability, sleeplessness, &c. are very different from those arising from the use of black tea. Their characteristic physical differences are well known; but they possess peculiar chemical properties to be noticed immediately, and which have always been ascribed by chemists to the effect of high temperature in the process of manufacture.

To explain these peculiarities, Mr. Warrington directs attention to certain changes which take place in herbs and vegetable substances dried in different modes. From observations made upon various substances of this kind, in the routine work of the establishment, to which Mr. Warrington is attached, especially the exsiccation of medicinal herbs, he had been led to form certain inferences as to changes taking places in these articles. These herbs are for the most part nitrogenous plants, as *Atropa belladonna*, *Hyoscyamus niger*, *Conium maculatum*, and others. The plants are brought to the establishment by the growers or collectors from the country, tied up in bundles, and when they arrive fresh and cool, they dry of a good *bright green* color; but on the contrary, it is found, that if they are delayed in their transit, or remain in a confined state for too long a period, they become heated, from a species of spontaneous fermentation, and when loosened and spread open, they emit vapors and are sensibly warm to the hand. When such plants are dried, the whole of the *green color* is found to have been destroyed; and a *red-brown* and sometimes a *blackish-brown* tint is the result. Mr. Warrington also noticed, that a clear infusion of such leaves, evaporated carefully to dryness, was not all undissolved by water, but left a quantity of brown *oxidized extractive matter*, to which the denomination of *Apothene* has been applied by some chemists. A similar result is obtained by the evaporation of an infusion of black tea.

The same action takes place upon the exposure of the infusions of many vegetable substances to the oxidizing influence of the atmosphere. They become darkened on the surface; and this gradually spreads through the solution, and on evaporation, the same *oxidized extractive matter* will remain insoluble in water.

Mr. Warrington further found that the green teas when wetted and re-dried, with exposure to the air, were nearly as dark in color as the ordinary black teas. From these observations, therefore, Mr. Warrington was led to form the conclusion, that the peculiar characters and chemical differences which distinguish black tea from green, were to be attributed to a species of heating and fermentation accompanied with oxidation by exposure to the air, and not to its being submitted to a high temperature in the process of drying, as has been generally believed. This opinion received some confirmation by ascertaining from parties conversant with the Chinese manufacture, that the leaves for the black teas were always allowed to remain exposed to the air, in mass, for some time before they were roasted.

Mr. Ball, in his work on the manufacture of tea, has described in detail the whole series of these processes, fully confirming the opinion previously formed by Mr. Warrington. Some of these facts had been published in Batavia in 1844, by Mr. Jacobson in the Dutch language. In the preface to his work Mr. Ball says: "It will be seen by dates incidentally adverted to, that the facts and most of the materials of this work, were established and collected thirty years ago."—"These facts, as well as other materials, were derived from conversation with growers and manipulators from the tea districts; from written documents furnished by Chinese; from published works in the same language diligently sought out; and also from correspondence with a Spanish missionary long resident in the province of Fokin. These were all put into their present form full twenty years ago, and were read to one or two friends during my residence in China."—"They were not, however, so arranged, with any view to immediate publication."—"They were thus disposed as the best mode of recording and keeping together the facts and materials I had collected."—"But it was not till the year 1844, when I received Mr. Jacobson's Handbook on the Cultivation of Tea in Java, that I found my own views so far confirmed, and my information such, as to justify me in bringing my labors to a close."

Of these facts the following is a summary.

The processes peculiar to the preparation of black tea, are styled Leang-Ching, To-Ching, annd Oc-Ching, and these all consist in carefully watched and regulated processes of *spontaneous heating* or *slow fermentation* of the leaves until a certain degree of fragrance is developed. The leaves are said to *wither and give*, and become soft and placid. The utmost care, practical skill, and experience is required in the properly conducting these operations; and as soon as the proper point is arrived at, the leaves are to be immediately removed to the Kuo or roasting-pan. After being roasted and rolled two or three times, they are then to be dried, and this is effected in the Poey-long, which consists of a cylinder of basket-work, open at both ends, and covered on the outside with paper; it is about 2½ feet in height and 1½ in diameter, which diameter is diminished in the centre like an ordinary dice-box to one foot and a quarter. This stands over and round a small charcoal fire, and is supplied with cross-bars about fourteen inches above the fire, on which an open sieve containing the tea is placed; and a small aperture about an inch and a half in diameter is made in the centre of the tea with the hand, so that an ascending current of air and the products of the combustion pass through and over the tea contained in the sieve. A circular, flat bamboo tray is passed partially over the mouth of this cylinder, and most probably serves to regulate the rapidity of the ascending current; prevent the admission of the cold air to the leaves, and at the same time allow a sufficient outlet for the generated watery vapors and the products of combustion. At the commencement of this operation, the moist leaves are still green and retain their vegetable appearance; after the drying has continued about half an hour, the leaves are turned, and again submitted to the heat for another half hour; they are then taken out, rubbed and twisted, and after sifting away the small dust, again returned to the sieve and drying tube. This operation of sifting is very necessary, to remove any of the small tea or dust which might otherwise fall through the meshes of the sieve on to the fire, and the products of their combustion would deteriorate and spoil the flavor of the tea. The leaves have now begun to assume their black color; the fire is diminished or deadened by ashes; and the operation of rolling, twisting, and sifting, is repeated once or twice until they have become quite black in color, well twisted, perfectly dry and crisp. They are then picked, winnowed and placed in large quantities over a very slow fire for about two hours, the cylinder being closed.

Now, that this black color is not owing to the fire is evident; for in cases mentioned by Mr. Ball, where the leaves have been dried in the sun, the same color is obtained; and on the other side, if roasted first, without the process of fermentation or *withering*, and then finished in the Poey-long, a kind of green tea is produced.

In the operations for the manufacture of green tea, on the contrary, the freshly-picked leaves are roasted in the Kuo at once, without delay, at a high temperature; rolled and roasted again and again, assisted sometimes with a fanning operation to drive off the moisture; and always with brisk agitation until the drying is completed.

The marked differences in the mode of manufacture of black and green tea, will, after what has been stated, fully account for all the variation of physical and chemical properties already mentioned.—*Quarterly Journ. Chem. Society.*

